# Reduction in Ochratoxin A Occurrence in Coffee: From Good Practices to Biocontrol Agents

**DOI:** 10.3390/jof10080590

**Published:** 2024-08-20

**Authors:** Claudia López-Rodríguez, Carol Verheecke-Vaessen, Caroline Strub, Angélique Fontana, Sabine Schorr-Galindo, Angel Medina

**Affiliations:** 1 Magan Centre of Applied Mycology, Cranfield University, Cranfield MK43 0AL, UKa.medinavaya@cranfield.ac.uk (A.M.); 2 Qualisud, University of Montpellier, CIRAD, Institut Agro, IRD, Avignon University, University of La Réunion, 34095 Montpellier, France; caroline.strub@umontpellier.fr (C.S.); angelique.fontana@umontpellier.fr (A.F.); sabine.galindo@umontpellier.fr (S.S.-G.)

**Keywords:** ochratoxin A, toxigenic fungi, post-harvest management, lactic acid bacteria, yeasts, biocontrol, biodegradation, detoxification

## Abstract

Ochratoxin A (OTA) is a mycotoxin mainly produced by *Aspergillus* section *Circumdati* and section *Nigri* across the coffee chain. OTA is nephrotoxic and is a threat to human health. This review summarizes current knowledge on how to reduce OTA concentration in coffee from farm to cup. After a brief introduction to the OTA occurrence in coffee, current good management practices are introduced. The core of this review focuses on biocontrol and microbial decontamination by lactic acid bacteria, yeasts and fungi, and their associated enzymes currently reported in the literature. Special attention is given to publications closest to in vivo applications of biocontrol agents and microbial OTA adsorption or degradation agents. Finally, this review provides an opinion on which future techniques to promote within the coffee supply chain.

## 1. Introduction

Coffee is the main drink consumed worldwide after water, with a 1.6-fold production increase in the last 20 years [1,2]. In 2022, the main producing region was South America (48.3%) followed by Asia and Oceania (29.6%) [2]. There are two main varieties of coffee, Arabica and Robusta, accounting for 56% and 44% of the worldwide production, respectively [2,3].

Coffee trade can be subjected to compliance with the maximum legal limits regarding Ochratoxin A (OTA) concentration. Structurally, OTA is a chlorinated isocoumarin compound, with a molecular weight of 403.813 Da, and is considered mutagenic, nephrotoxic, and potentially carcinogenic for humans (group 2B by the International Agency for Research on Cancer) [4]. OTA is a secondary metabolite produced by the fungal genera *Aspergillus* and *Penicillium*. The United States Food and Drug Administration (FDA) and Codex Alimentarius has no set limits for OTA levels in coffee, while the European Commission has set maximum limits of 3 µg/kg for roasted coffee beans and 5 µg/kg for soluble coffee [5]. The OTA occurrence in coffee beans is generally produced by fungi belonging to the genus *Aspergillus*.

Hereafter, we will present the occurrence of OTA contamination in the coffee supply chain and summarize recent efficient strategies to prevent, control, and/or remove OTA contamination in coffee. Good management practices (GMP) are then introduced, and the research gaps to address in OTA biocontrol research in coffee are highlighted to promote future application.

### 1.1. Ochratoxin A Contamination throughout the Coffee Supply Chain

**Figure 1:**
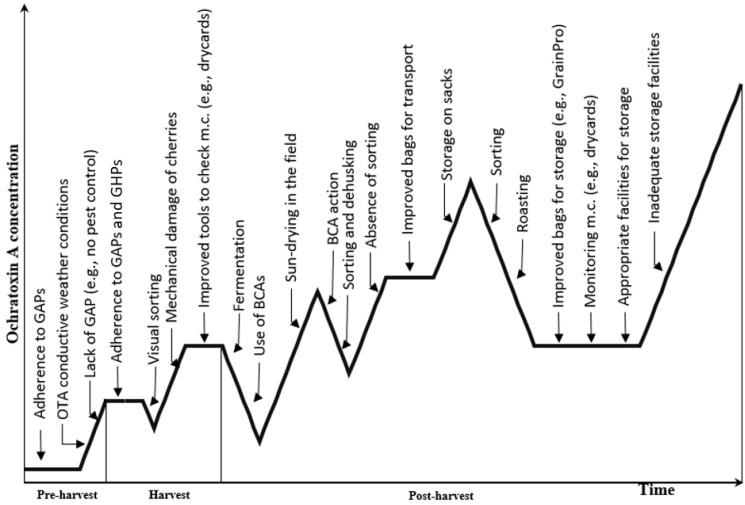
Time course of ochratoxin A formation in relation to proposed mitigation strategies in coffee during pre-harvest, drying, and post-harvest (adapted from Pitt et al., 2013) [9]. M.c.: moisture content; GAP: good agricultural practices; BCA: biocontrol agent.

Ochratoxin A contamination can occur from farm to cup (Figure 1). Contamination with *Aspergillus* spp. can begin at pre-harvest, especially at the flowering stage through the carriage of spores by coffee berry borers (*Hypothenemus hampei*) and delayed harvest due to overripen cherries [6]. At pre-harvest, after contamination with the spores, optimal weather conditions of temperature and water activity, as specified in Table 1, trigger their germination. However, it is at post-harvest, during drying, that most of the fungal contamination occurs [7]. Good post-harvest management practices (GPMP) help reduce fungal growth, OTA production, and facilitate the development of competitive micro-organisms [8].

**Figure A1:**
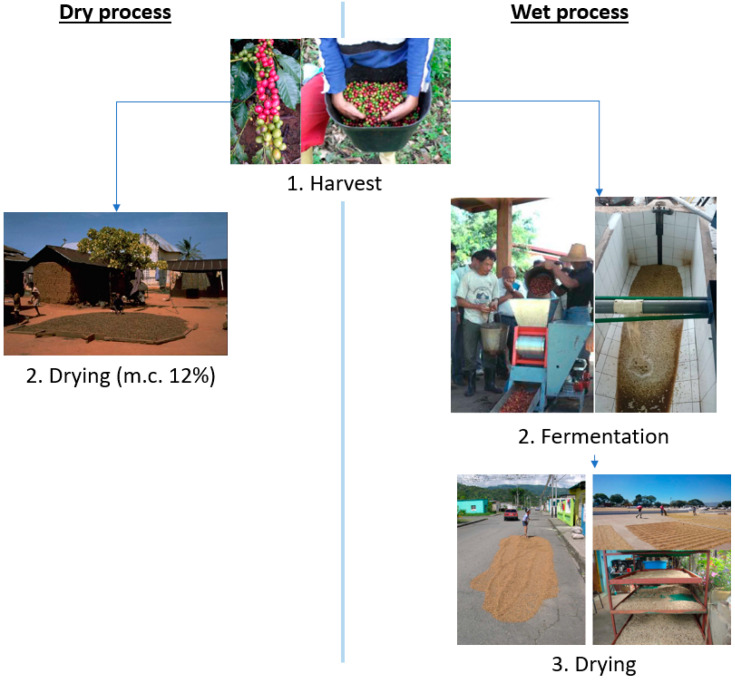
Images of the different steps of the dry and wet processes. Photos provided by CIRAD.

During post-harvest, coffee cherries can go through two main processes: natural (dry) and washed (wet) methods, detailed in Figure A1 in Appendix A [16]. In the dry method, coffee is first sorted and then sun-dried. During the wet method, coffee is fermented in water and then sun-dried, giving parchment coffee as the final product (without mucilage) [17]. Good hygiene practices (GHP) including disinfection are paramount to avoid equipment contamination with mycotoxigenic fungi [18,19].

The drying step can be divided into three phases. The first phase lasts between six and eight days and is dominated by harmless hydrophilic micro-organisms, mainly bacteria, due to the high water activity (a_w_; 0.95 a_w_) of the coffee beans. The transition phase (0.90 a_w_) lasts around six days. This phase facilitates the growth of mesophilic and xerophilic micro-organisms, such as yeasts and fungi, and it will be during this phase that ochratoxigenic fungi may produce OTA, especially in the dry method, where cherries are dried with the pulp. At the last step, when coffee cherries reach a moisture content of 12% or 0.60 a_w_, they are ready for long-term storage. In the case of drying after wet processing, the total length of drying will be reduced from 12 for dry processing to 7 days, reducing the risk of contamination [16,20].

### 1.2. Ochratoxin A Decontamination Strategies

Climate change, fungicide resistance, and net-zero-targeted policies are pushing towards resilient and natural methods to reduce OTA contamination in coffee [21]. Some biocontrol micro-organisms such as bacteria, yeasts, and atoxigenic fungi or their metabolites have been shown to prevent and reduce OTA concentration in coffee [22]. Biocontrol agents (BCAs) have different strategies to prevent filamentous fungi from growing and/or producing mycotoxins. Some of the strategies to prevent growth are the production of anti-germinative compounds, chitinase activity or production of Volatile Organic Compounds (VOCs), as well as competition for nutrients and space. These BCAs can also directly prevent the production via the inhibition of OTA biosynthesis [23].

Another approach is the reduction in OTA concentration once they are formed through biological, (adsorption or degradation of mycotoxins), physical (radiation, temperature, or ultrasounds) and chemical methods (ozone or adsorbents) [24]. Although, once OTA is produced, it is very hard to remove due to its heat stability. During roasting, the reduction in OTA can widely vary from 12 to 90%, depending on the roasting temperature and the initial OTA contamination [25].

BCA-based solutions have several advantages in comparison to the physical and chemical methods since they can be very specific, efficient, and environmentally friendly, as well as preserving or improving the organoleptic and nutritive quality of coffee [26]. Such BCA solutions should be generally recognized as safe, and not generate toxic OTA degradation by-products [27,28].

## 2. Occurrence and Contamination of Ochratoxin A in Coffee

### 2.1. Ochratoxin A Producers and Optimum Conditions of Production

The main OTA producers in coffee belong to *Aspergillus* sections *Circumdati* and *Nigri* [20]. In section *Circumdati*, *A. ochraceus*, *A. westerdijkiae* and *A. steynii* produce OTA, with *A. westerdijkiae* and *A. steynii* being the highest producers. In section *Nigri*, *A. carbonarius* and *A. nigri*, are producers, with *A. carbonarius* being the highest of both [14]. The optimal ranges of a_w_ and temperature (T; °C) for the germination, growth, and toxin production of these fungi are detailed in Table 1. This table shows that, for *A. carbonarius*, spore germination and optimal growth are at higher temperatures than the other three fungi. However, OTA production is optimal at higher temperatures for the species *A. westerdijkiae* and *A. steynii*. These ranges should be considered throughout all coffee chains as conditions to avoid [29].

### 2.2. Occurrence of Ochratoxin A in Coffee

In the last 26 years, the concentration of OTA has been detected in 27 countries around the world (Table 2). Pardo et al. (2004) [11] analyzed 57 green coffee samples (41 of Arabica Coffee and 16 from Robusta) from America, Asia, and Africa. The OTA concentration results ranged between 0.1 and 80 µg/kg, and the average was 6.7 µg/kg, 2-fold above the European Commission’s (EC) regulatory limit for roasted coffee beans [5]. From the 57 samples, 12 were African samples, with results ranged between 2.4 and 23.3 µg/kg, and the average was 10.9 µg/kg, 3-fold higher than the EC’s regulatory limit. A meta-analysis from Khaneghah et al. (2019) [30], based on 36 published articles, studied the prevalence and concentration of OTA in different countries. In the cited article, they found that higher poverty, a lower Human Development Index, and increasing annual rainfall significantly increase the prevalence and concentration of OTA in coffee.

In the present review, we analyzed the median, minimum, and maximum level of OTA in coffee from different origins and markets (Table 2). We found that Asia’s OTA level has a higher median than other coffee-producing continents such as Africa or Central and South America. However, the maximum values of OTA were found in African coffees. Central and South America were the coffee-producing areas with less OTA contamination, although the three producing areas showed maximum OTA levels above the EC’s regulations. Potentially due to the enforcement of the EC’s regulations, the OTA levels in the European market (Table 2) were never above the EC’s limits. It is important to emphasize that the number of samples analyzed was the highest in Europe (808) and the lowest in Africa (33), which highlights the need for more OTA quantification in coffee on this continent.

Surprisingly, the comparison of OTA levels between green, roasted, and soluble coffee showed similar levels in green and roasted coffee. This could suggest a moderate capacity of roasting to reduce OTA concentration and highlight the need of implementation of BCA-based strategies. Robusta coffee had a slightly higher level of OTA compared to Arabica; this is probably because the dry process is more used for Robusta [14,31,32].

**Table 2:** OTA levels found in different coffees from 1997 to 2023.

Country	Coffee Stage	Coffee Species	No Positive/Total	Average (µg/kg)	Range (µg/kg)	Fungi	Year	Reference
Asia’s coffee-producing countries and markets								
NS ^1^ Asiatic countries	Green	NS ^1^	NS/14	6	1.6–31.5	NS	NS	[11]
India	Green	Robusta dry	2/2	0.12	0.1–0.14	NS	NS	[33]
India	Roasted	Arabica	1/1	2.3	ND ^2^	NS	NS	[34]
India	Roasted ground	Robusta	1/1	0.79 ± 0.11	ND	NS	NS	[35]
Saudi market	Roasted	NS	4/89	3.4	2.2–5.6	NS	2015–2019	[36]
Saudi market	Green	NS	28/206	7.3	1.2–50	NS	2015–2019	[36]
Thailand	Roasted	Arabica	1/1	0.39	ND	*A. westerdijkiae*	February2018	[7]
Vietnam	NS	Arabica	1/3	2.3	ND	*A. carbonarius*, *A. westerdijkiae*, *A. niger*	NS	[37]
Vietnam	NS	Robusta	1/13	6.3	ND	*A. carbonarius*, *A. niger*	NS	[37]
Vietnam	Roasted	Robusta	1/1	1.5	ND	NS	NS	[34]
Vietnam	Green	Robusta dry	9/9	2.54	0.64–8.05	NS	NS	[33]
Vietnam	Roasted ground	Robusta	1/1	1.56 ± 0.33	ND	NS	NS	[35]
			No positive/Total samples in Asia: 18/46	Median: 2.3 µg/kg	Max: 6 µg/kg; Min: 0.12 µg/kg			
Africa’s coffee-producing countries								
NS African countries ^1^	Green	NS	NS/12	10.9	2.4–23.3	NS	NS	[11]
Cameroon	Green	Arabica wet	0/2	ND	ND	NS	2017	[38]
Cameroon	Green	Robusta dry	4/4	0.52	0.1–0.7	NS	2017	[38]
Cameroon	Green	Arabica dry	1/2	0.1	ND–0.1	NS	2017	[38]
Cameroon	Roasted	Robusta	1/1	2.3	ND	NS	NS	[34]
Ethiopia	Roasted	Arabica	1/1	2	ND	NS	NS	[34]
Ivory Coast	Green	Robusta dry	54/54	88.26	0.55–760.24	*Aspergillus carbonarius*	2020	[32]
Ivory Coast	NS	Robusta	5/5	7.03	+/− 0.726	*Aspergillus* section Nigri	2008 and 2009	[39]
Ivory Coast	Roasted	Robusta	1/1	2.3	ND	NS	NS	[34]
Uganda	Roasted ground	Robusta	1/1	1.51 ± 0.10	ND	NS	NS	[35]
Uganda	Green	Robusta dry	2/2	0.295	0.28–0.31	NS	NS	[33]
			No positive/Total samples in Africa: 16/33	Median: 2 µg/kg	Max: 10.9 µg/kg; Min: 0.1 µg/kg			
America’s coffee-producing countries and markets								
NS American countries	Green	NS	NS/31	5.4	1.3–27.7	NS	NS	[11]
Argentine market	Green	NS	5/5	5.77	0.20–20.3	NS	NS	[40]
Argentine market	Roasted ground	NS	13/24	1	0.11–5.78	NS	NS	[40]
Argentine market	Soluble	NS	17/22	1.99	0.22–13.66	NS	NS	[40]
Brazil	Roasted	NS	5/16	ND	0.9–9	NS	NS	[41]
Brazil	Roasted ground	Arabica	1/1	ND	ND	NS	NS	[35]
Brazil	Green	Arabica	9/40	2.45	0.47–4.82	NS	NS	[42]
Brazil	Roasted	Arabica	1/1	1.4	ND	NS	NS	[34]
Brazil	Roasted ground	NS	23/34	0.9	0.3–6.5	NS	NS	[43]
Brazil	Soluble	NS	8/16	2.2	0.5–5.1	NS	NS	[43]
Brazil	Green	Arabica dry	5/11	0.2	0.01–1.63	NS	NS	[33]
Canada market	Roasted ground	NS	38/59	0.4	0.1–2.3	NS	NS	[44]
Canada market	Soluble	NS	15/21	0.8	0.1–3.1	NS	NS	[44]
Chile market	Roasted	NS	24/24	0.47 ± 0.20	0.30–0.84	NS	NS	[45]
Chile market	Soluble	NS	39/39	1.8 ± 1.81	0.28–5.58	NS	NS	[45]
Colombia	Roasted ground	Arabica	1/1	ND	ND	NS	NS	[35]
Colombia	Green	Arabica wet	3/3	0.57	0.08–0.12	NS	NS	[33]
Costa Rica	Green	Arabica wet	4/9	0.042	0.02–0.12	NS	NS	[33]
Costa Rica	Roasted	Arabica	1/1	1.5	ND	NS	NS	[34]
Honduras	Roasted ground	Arabica	1/1	0.5 ± 0.04	ND	NS	NS	[35]
Mexico	Roasted	Arabica wet	27/77	0.36 ± 0.62	0.05–148.62	NS	2017–2018	[46]
Mexico	Green	Arabica wet	5/7	0.60 ± 0.55	0.05–12.50	NS	2017–2018	[46]
Mexico	Roasted	Robusta dry	10/12	0.37 ± 0.63	0.05–3.4	NS	2017–2018	[46]
Mexico	Green	Robusta dry	7/7	0.70 ± 0.60	0.05–3.4	NS	2017–2018	[46]
Mexico	Instant	NS	10/13	0.93 ± 0.71	0.05–1.37	NS	NS	[46]
Peru	Roasted ground	Arabica	1/1	0.51 ± 0.07	ND	NS	NS	[35]
Puerto Rico	Roasted ground	Arabica	1/1	0.86 ± 0.50	ND	NS	NS	[35]
Sao Tome and Principe	Roasted ground	Arabica	1/1	0.6 ± 0.19	ND	NS	NS	[35]
Santos	Roasted	Arabica	1/1	1.4	ND	NS	NS	[34]
			No positive/Total samples in America: 217/363	Median: 1 µg/kg;	Max: 5.4 µg/kg; Min: 0.042 µg/kg			
European markets								
France	Roasted ground	NS	30/30	1.1	0.025–11.9	NS	NS	[47]
Germany	Green	NS	22/82	1.29	0.23–24.5	NS	NS	[48]
Germany	Roasted	NS	191/419	0.99	12.1–0.21	NS	NS	[48]
Germany	Soluble	NS	29/41	1	4.8–0.28	NS	NS	[48]
Hungary	NS	NS	33/50	0.57	0.17–1.3	NS	NS	[49]
Italy	Roasted	NS	8/30	1.03 ± 0.17	ND	NS	NS	[50]
Portugal	Roasted	NS	2/6	1.84 ± 0.03	10.31–0.71	NS	NS	[35]
Portugal	Roasted ground	NS	1/5	1.45 ± 0.02	ND	NS	NS	[35]
Spain	Roasted	NS	43/45	2.07 ± 0.61	1.30–5.24	NS	NS	[51]
UK	Roasted	NS	17/20	0.6	0.2–2.1	NS	NS	[52]
UK	Soluble	NS	64/80	1	0.1–8	NS	NS	[52]
			No Positive/Total samples in Europe: 440/808	Median: 1.03 µg/kg;	Max: 2.07 µg/kg; Min: 0.57 µg/kg			

^1^ NS: Not specified. ^2^ ND: Not determined.

## 3. Strategies to Reduce Ochratoxin A Occurrence in Coffee

### 3.1. Good Management Practices for Ochratoxin A Prevention at Post-Harvest

Figure 1 represents the time course of OTA formation from the pre- to post-harvest stages. Fermentation, drying, or storage are the critical steps to prevent ochratoxigenic fungi growth. Fermentation is used to limit oxygen availability and encourage harmless, competitive micro-organisms while degrading the mucilage. It is recommended to stop the fermentation before the mucilage is removed and to dry the coffee [20]. The drying should be performed evenly to avoid moisture content (m.c.) or a_w_ above 12% or 0.60, respectively [53]. Moreover, techniques such as the Collapsible Dryer Case™ (GrainPro Inc., Washington, DC, USA) will help to avoid direct contact with the soil, which is a source of contaminants [54]. Other scenarios could include concrete ground or a bamboo table, which have been proven to prevent fungal contamination [7].

During drying and storage, the m.c. should be monitored [55]. Cervini et al. (2022) suggested the use of DryCard™, developed by the University of California Davis (UC Davis). This tool would help to monitor the % of Relative Humidity (% RH) during drying and storing [56]. Before storage, coffee should be manually sorted and dehusked to remove the fractions most contaminated with OTA [20]. Coffee is normally stored in jute sacks, which tend to be reused, representing a critical factor for post-harvest contamination with spoilage and mycotoxigenic fungi [53]. The Purdue Improved Crop Storage (PICS) bags are a hermetic storage technology consisting of two inner layers of polyethylene protected by an outer layer with a woven polypropylene bag. PICS bags have been previously successfully used in peanuts to reduce weight loss and pest contamination [57]. Other bags like the GrainPro SuperGrain bag or GrainPro Safeliner [53] can also maintain the a_w_ below 0.60 for up to 27 days after drying, preventing OTA contamination during storage and transportation.

According to the Codex Alimentarius (2009) [55], the best conditions to prevent OTA during transportation and storage are low temperature (3–7 °C) and a a_w_ ≤ 0.60 to avoid rewetting of the beans [55]. Overall, the application of risk management metrics at the key steps in coffee post-harvest is a useful tool for managers to determine the suitability of a coffee batch for a target market [58].

In addition to GMP, there are complementary methods to efficiently reduce contamination in coffee such as the application of biocontrol agents. Hereafter, microbiological strategies will be presented, with a special focus on LAB, yeasts, and fungi isolated from coffee.

### 3.2. Biological Strategies for Ochratoxin A Prevention in Coffee

One of the strategies to reduce OTA concentration in coffee is the use of BCAs. Depending on the BCA, the action mechanisms involved could be the production of antimicrobial compounds, inhibition of biosynthetic pathways or gene clusters, competition for space and nutrients and/or physical adhesion to the mycelium or to the spores. These mechanisms can reduce spores’ germination, fungal growth, and/or OTA production. Different bacteria and yeasts have been used as BCAs and the conditions of study are summarized in Table 3 and Table 4. Section 3.2.3 summarizes the fungal BCAs tested against OTA occurrence. In Section 3.2.4, we provide an overview on the current state of the art of combined BCA application and the potential for commercialization.

#### 3.2.1. Bacteria

Within the bacteria domain, the groups more often described as decontaminating are the order lactic acid bacteria (LAB) and the phylum Actinobacteria. LAB have been largely used as antifungals in food. Some examples of LAB with activity against the growth, sporulation, and spore germination of ochratoxigenic fungi are shown in Table 3. The most tested LAB against different strains of ochratoxigenic fungi (i.e., *A. carbonarius*) are *Lactiplantibacillus* spp. *L. plantarum* [59,60]. Djossou et al. (2011) isolated from coffee two strains of *L. plantarum* with the ability to inhibit *A. carbonarius* growth from 60 to 100% [61]. Other species such as *Lactiplantibacillus brevis* was identified for its capacity to reduce OTA production by 91% through the down-regulation of the OTA biosynthetic genes in *A. carbonarius* [62].

The reduction in fungal growth can be linked to the production of metabolites such as organic acids, carbon dioxide, hydrogen peroxide, phenyl lactic acid (PLA), and bioactive low molecular weight peptides [59]. The organic acid produced by LAB in the highest quantity is lactic acid [63]. PLA has been proved to delay the growth of *Aspergillus* and *Penicillium* spp. However, high concentrations of PLA of 50 mM are needed as the Minimum Inhibitory Concentration (MIC), while LAB naturally produce a much lower concentration, between 0.1 and 1.6 mM. A solution is the addition of phenyl pyruvic acid (PPA) during LAB growth on MRS to enhance the PLA production. The use of PPA remains uncertain due to potential safety issues.

Other orders (i.e., Bacillales) or phyla (i.e., Actinobacteria) have been studied for their OTA presentation capacity. An example is the work by Shang et al. (2019) [63], who isolated the strain *Bacillus megaterium* from soil, which had the capacity to reduce (in solid media) *A. ochraceus* mycelial growth by 41.9%. Other Bacillus species such as *B. amyloliquefaciens* or *B. subtilis* have been reported to reduce 96 and 94% of OTA in confrontation with *A. westerdijkiae* [64]. 

**Table 3:** Cultures of LAB tested in various conditions for their efficacy to reduce growth, spore germination, or OTA production against different OTA-producing fungi isolated from coffee. CFS = Cell Free Supernatant, GCM = green coffee media.

Name	Media Used	Inoculum (CFU/mL)	[Fungal] (sp/mL)	OTA Reduction Efficacy (%)	Incubation Period	Environmental Conditions	Incubation Condition	Reference
*Bacillus amyloliquefaciens* RP103	GCM	10^9^	5 mm agar plug	96.1%	10 days	25 °C	Against *A. westerdijkiae*	[64]
*Bacillus subtilis* RP242	GCM	10^9^	5 mm agar plug	94.9%	10 days	25 °C	Against *A. westerdijkiae*	[64]
*Bacillus safensis* RF69	GCM	10^9^	5 mm agar plug	62.1%	10 days	25 °C	Against *A. westerdijkiae*	[64]
*Lactobacillus plantarum*	MRS/CYA double layer	NS ^1^	10^4^	2 cm inhibition fungal growth	72 h	25 °C	Against *A. carbonarius*	[61]
*Lactobacillus brevis 8-2B isolated from grapes*	PDA	10^7^	10^7^	91%	11 days	28 °C	Against *A. carbonarius* from grapes	[62]
*L. brevis* 8-2B	PDB/PDA	CFS of 10^5^	10^5^	Inhibition of spore germination	24 h	28 °C	Against *A. carbonarius*	[62]
*L. brevis* 8-2B	PDB	10^7^	10^7^	Inhibition of spore germination	5 h	180 rpm, 28 °C	Against *A. carbonarius*	[62]
*L. brevis* LPBB03	MRS	10^6^	10^6^	50% growth inhibition	5 days	28 °C	Against *A. westerdijkiae*	[65]

^1^ NS: Not specified.

#### 3.2.2. Yeasts

The most widely studied species is *Saccharomyces cerevisiae.* The strain *S. cerevisiae* CCMA 0159 has been recognized for its biocontrol properties against *A. carbonarius*, *A. ochraceus*, *and A. westerdijkiae* [66]. During this study, confrontations at temperatures between 18 and 38 °C and a_w_ between 0.91 and 0.99 a_w_ in a coffee-based medium were analyzed. The results showed that *S. cerevisiae* significantly reduced contamination with OTA by 76% and growth by 96% of the species *A. carbonarius* at 28 °C and 0.99 a_w_, which were the optimum conditions for yeast growth (Table 4). The possible mechanism used by the yeast to reduce OTA and growth was substrate competition.

According to Lino de Souza et al. (2020) [67], this same strain of *S. cerevisiae* can reduce OTA production through VOCs. However, in the in vivo experiments at a pilot scale (using 10 kg of coffee cherries as substrate), the inoculation of the *S. cerevisiae* CCMA 0159 and *A. carbonarius* before drying and until coffee reached 11–12% of the m.c. did not reduce the OTA concentration in the final dried coffee. These results might be due to the environmental conditions, since the temperature during the pilot scale was 18.2 °C, which allowed for the growth of *A. carbonarius* but was too low for the yeast [68]. This highlights the importance of testing all potential BCAs in field conditions.

Besides *S. cerevisiae*, there are many other yeasts species with potential BCA properties. For example, Masoud and Kaltoft, (2006) isolated three strains of yeasts, *Pichia anomala*, *P. kluyveri*, and *Hanseniaspora uvarum*, which reduce growth by at least 50% and 100% of OTA biosynthesis via *A. ochraceus* on Malt Extract Agar (MEA). However, in coffee media, only *P. anomala* and *P. kluyveri* were able to reduce growth by 50% and 95%, respectively, and 100% of OTA production [69]. This observation highlights the significant influence of incubation media on experimental outcomes, emphasizing the importance of exhaustive parameter assessment. Systematically evaluating an array of different media, including synthetic and coffee-based media, is key in elucidating the mechanisms behind biocontrol and in targeting the most suited BCA for application in coffee post-harvest.

Also, complementary tests on yeast glucose peptone broth indicated that the yeast-free supernatant completely reduced the germination of the spores, via the depletion of glucose, and the production of VOCs such as ethyl acetate.

De Melo Pereira et al. (2016) isolated the yeast strain *P. fermentans* LPBYB13 from coffee and showed an inhibition of *A. westerdijkiae* growth by producing an inhibitory halo of > 0.5 cm against the fungus on synthetic media in vitro [65]. In vivo, this strain was confirmed to reduce OTA production by up to 88% in coffee beans. The suspected mechanisms used by this yeast might be the competition for nutrients and space or the production of extra-cellular toxic metabolites [70]. Another mechanism used by the yeasts is the adhesion to the mycelium to reduce fungal growth, as shown in the Scanning Electron Microscopy (SEM) photos, where *Rhodosporidiobolus ruineniae* adheres to the wall of the mycelium, reducing its turgidity and growth, and also reducing OTA production by 58.3% [71]. Similarly, Lino de Souza et al. (2020) observed, through SEM, the presence of a biofilm of *S. cerevisiae* cells adhered to the coffee husk, which is a mechanism responsible for the reduction in OTA production. 

**Table 4:** Cultures of yeasts tested in various conditions for their efficacy to reduce growth, spore germination, or OTA production against different OTA-producing fungi isolated from coffee. YPD = Yeast Peptone Dextrose; PDA = Potato Dextrose Agar; MEA = Malt Extract Agar; YEG = Yeast Extract and Glucose liquid medium; CFS = Cell Free Supernatant, TLC = thin layer chromatography; GCM = green coffee media; RH = Relative Humidity.

Name	Media Used	Inoculum (Cells/mL)	[Fungal] (sp/mL)	OTA Reduction Efficacy (%)	Incubation Period	Environmental Conditions	Incubation Condition	Reference
*C. friedrichii* 778	Sealed plates YPD/PDA	10^8^	10^7^	99.9%	7 days	25 °C	Against *A. carbonarius*	[70]
*C. friedrichii* 778	Sealed plates YPD/PDA	10^8^	10^7^	99.9%	7 days	25 °C	Against *A. ochraceus*	[70]
*Candida intermedia* 235	Sealed plates YPD/PDA	10^8^	10^7^	99.9%	7 days	25 °C	Against *A. carbonarius*	[70]
*C. intermedia* 235	Sealed plates YPD/PDA	10^8^	10^7^	99.9%	7 days	25 °C	Against *A. ochraceus*	[70]
*Cyberlindnera jadinii* 273	Sealed plates YPD/PDA	10^8^	10^7^	99.9%	7 days	25 °C	Against *A. carbonarius*	[70]
*C. jadinii* 273	Sealed plates YPD/PDA	10^8^	10^7^	99.9%	7 days	25 °C	Against *A. ochraceus*	[70]
*Hanseniaspora uvarum*	MEA/CA	10^6^	10^6^	OTA not found by TLC	7 days	30 °C	Against *A. ochraceus*	[69]
*Lachancea thermotolerans* 751	Sealed plates YPD/PDA	10^8^	10^7^	99.9%	7 days	25 °C	Against *A. carbonarius*	[70]
*L. thermotolerans* 751	Sealed plates YPD/PDA	10^8^	10^7^	99.9%	7 days	25 °C	Against *A. ochraceus*	[70]
*Pichia anomala* S12	MYGP broth	10^6^	10^6^	Spore germination inhibition	72 h	30 °C	Against *A. ochraceus*	[69]
*P. anomala* S12	MYGP broth	CFS 10^6^ cells/mL	10^6^	Spore germination inhibition	72 h	30 °C	Against *A. ochraceus*	[69]
*P. anomala* S12	MEA/CA	10^6^	10^6^	50% growth inhibition	7 days	30 °C	Against *A. ochraceus*	[69]
*P. anomala* S12	MEA/CA	10^6^	10^6^	TLC, OTA not found	7 days	30 °C	Against *A. ochraceus*	[69]
*Pichia kluyveri*	MEA/CA	10^6^	10^6^	TLC, OTA not found	7 days	30 °C	Against *A. ochraceus*	[69]
*Pichia fermentans* LPBYB13	YM	10^6^	10^6^	50% growth inhibition	5 days	28 °C	Against *A. westerdijkiae*	[65]
*P. fermentans* LPBYB13	Coffee beans	10^6^ CFU/g	10^6^ sp/g	88%	10 days	28 °C	Against *A. westerdijkiae*	[65]
*Saccharomyces cerevisiae* CCMA 0159	GCM	NS ^1^	10^5^	76%	10 days	0.99 a_w_, 28 °C	Against *A. carbonarius*	[66]
*S. cerevisiae* CCMA 0159	GCM	NS	10^5^	96%	10 days	0.99 a_w_ 28 °C	Against *A. westerdijkiae*	[66]
*S. cerevisiae* CCMA 0159	GCM	NS	10^5^	95%	10 days	0.99 a_w_, 28 °C	Against *A. ochraceus*	[66]
*Saccharomyces cerevisiae*	Arabica wet-processed coffee	10^8^	NS	100%	10 min	RH 90%, 17–36 °C	NS	[72]
*S. cerevisiae*	Arabica wet-processed coffee	10^8^	10^7^	96%	10 min	RH 90%, 17–36 °C	Against *A. ochraceus*	[72]
*S. cerevisiae*	Arabica dried-processed coffee	10^8^	NS	78.2%	10 min	RH 90%, 17–36 °C	NS	[72]
*S. cerevisiae*	Arabica dried-processed coffee	10^8^	10^7^	92.8%	10 min	RH 90%, 17–36 °C	Against *A. ochraceus*	[72]
*S. cerevisiae*	Robusta wet-processed coffee	10^8^	NS	100%	10 min	RH 90%, 17–36 °C	NS	[72]
*S. cerevisiae*	Robusta wet-processed coffee	10^8^	10^7^	66.7%	10 min	RH 90%, 17–36 °C	Against *A. ochraceus*	[72]
*S.s cerevisiae*	Robusta dried-processed coffee	10^8^	NS	75%	10 min	RH 90%, 17–36 °C	NS	[72]
*S. cerevisiae*	Robusta dried-processed coffee	10^8^	10^7^	71.4%	10 min	RH 90%, 17–36 °C	Against *A. ochraceus*	[72]
*Rhodosporidiobolus ruineniae*	YEG	10^7^	10^7^	58.3%	6 days	25 °C	Against *A. carbonarius*	[71]

^1^ NS: Not specified.

#### 3.2.3. Non-Mycotoxigenic Fungi

De Almeida et al. (2019) isolated eight non-mycotoxigenic strains from coffee: *Rhizopus* spp. (two isolates), *Lichtheimia sp.* (one isolate), and *Aspergillus* spp. (five isolates). All showed an in vitro capacity to reduce the growth and OTA production of the ochratoxigenic strains *A. ochraceus*, *A. westerdijkiae*, *A. carbonarius*, and *A. niger* on a Sabouraud medium. All strains except *Lichtheimia ramosa* reduced growth by 70%. OTA production of *A. ochraceus* and *A. niger* was completely inhibited by *L. ramosa* and *Aspergillus* sp. for the first, and by the non-toxigenic strain *A. niger* for the latter [73]. Although the action mechanisms are not well explained in the existing examples of non-mycotoxigenic fungi used as OTA BCAs in coffee, the literature considers important mechanisms during confrontations of non-toxigenic and mycotoxigenic fungi niche exclusion and the rate of utilization of nutritional sources [22].

#### 3.2.4. Commercialization of BCAs

In the coffee market sector, BCAs against OTA is a concept yet to be known. Instead, they are more familiarized with the concept of a starter culture. A starter culture is a set of micro-organisms added before fermentation (for wet-processed coffee) or before drying (dry-processed coffee). For coffee, the only commercially available starter culture with antifungal properties is provided by Lallemand Inc. (Montreal, QC, Canada) in the form of a dried yeast powder (LALCafe Oro™, Intenso™, Briosa™, Cima™, BSC™, manufactured by Lallemand Inc., Montreal, QC, Canada) [74]. It must also be noted that Velmourougane et al. (2011) [72] used, for the first time, a commercial baker’s yeast procured from a local bakery (*S. cerevisiae)* during the fermentation of parchment and cherries of Arabica and Robusta coffee. They confirmed, in vivo, the capacity of the yeast to reduce the OTA contamination in both types of coffee and processing, obtaining an OTA reduction between 66 and 96% after inoculating the coffee with the yeast and *A. ochraceus* (Table 3). The addition of LAB in a starter culture for coffee has not yet been commercialized.

However, it is also important to check the capacity of the BCAs to detoxify the OTA once produced. Hereafter, we will summarize the OTA adsorption and degradation mechanisms of different micro-organisms on synthetic or coffee-based matrices.

### 3.3. Biological Strategies to Reduce Ochratoxin A in Coffee

There are two biological strategies or mechanisms to reduce OTA concentration in coffee: adsorption and degradation. Adsorption consists of the binding of OTA produced by the mycotoxigenic fungi to the cell wall of the detoxifying micro-organism. The strength of this bond depends on the composition of the cell wall, but it is normally weak; thus, this mechanism is reversible, and the OTA is normally liberated back to the substrate. On the other hand, degradation is mediated through enzymes produced by the detoxifying micro-organism that breaks the structure of the OTA into smaller metabolites. In the next sections, the structural differences between LAB, yeasts and fungi, and their influence on the capacity to detoxify OTA will be presented and discussed.

#### 3.3.1. Biological Adsorption of Ochratoxin A by LAB

Adsorption is the processus of a chemical compound, such as OTA, being bonded to a surface. Mycotoxin adsorption by the cell wall of LAB is the most common mechanism of mycotoxin removal in media or food [28]. The cell wall of LAB is composed of a cytoplasmic membrane with polysaccharides, proteins, lipoteichoic acids, and teichoic acids attached. The adsorption of OTA by the cell wall of LAB is mainly linked to the presence of polysaccharides and peptidoglycans. Differences in the OTA binding capacity are specially influenced by the structure of the peptidoglycans and the number of available binding sites. Peptidoglycans are composed of linear glycan strands of *N*-acetyl glucosamine (GlcNAc) and *N*-acetyl muramic acid (MurNAc), which are interconnected by short peptides. The variable length of these peptides modulates the structure of peptydoglycans among different LAB strains [59]. Also, OTA binds to bacteria by non-covalent interactions such as hydrophobic or ionic interactions and hydrogen bonds [28,75]. OTA binding capacity is a phenomenon not yet well understood and depends on different factors such as the initial concentration of mycotoxins, bacterial growth phase and fraction of the cell, LAB cell number, LAB strain, growth media, the complexity and pH of the food, the incubation temperature, and the hydrophobicity of the bacterial cell wall [28,59,76].

Acid and heat treatment of the LAB cell walls are strategies to increase their binding capacity, which could be linked to an extension of the peptidoglycan net. Zhang et al. (2022) analyzed the ability to remove OTA on PDB by *Bacillus velezensis* E2 cell pellets and heat-treated cell pellets. Their results suggested that cell pellets and heat-treated cell pellets can adsorb, respectively, 68 and 94% of the initial OTA. This adsorption mechanism was boosted in the heat-treated cell pellets, probably due to the extended pores of the peptidoglycan layers after heat treatment [77]. Another example was reported by Shang et al. (2019) [78], where heat inactivation increased the binding of OTA to the wall of *Bacillus megaterium* by 15%.

The pH has an important role in modifying hydrophobic and electrostatic interactions between OTA and the LAB cell wall. It has been reported that pH 5 was optimal for the removal of OTA from MRS by the strain *Lactobacillus acidophilus* VM20, while at higher pH of 6, 7, or 8, the reduction in OTA was significantly lower [79]. It is also important to mention the role of the hydrophobicity of the bacterial cell wall, since *E. coli*, a Gram-negative bacterium is not capable of removing mycotoxins due to its moderately hydrophilic nature [28].

The stability of the mycotoxin–LAB complex is reversible and depends on the pH and environmental conditions but also on the bacterial strain and number of cells, for which there is correlation between the initial bacterial count and mycotoxin binding [35]. Not only the cell itself, but also the metabolites produced by LAB can bind to the OTA. These metabolites can be acids, phenolic compounds, fatty acids, reuterin, and low molecular weight bioactives [76].

#### 3.3.2. Biological Adsorption of Ochratoxin A by Yeasts

Ul Hassan et al. (2021) [80] analyzed the ability of four yeasts’ species to bind OTA to the cell walls: *Cyberlindnera jadinii 273*, *Candida friedrichii 778*, *Candida intermedia 235* and *Lachancea thermotolerans 751*, and *S. cerevisiae*. The strains presented a reduction efficacy of OTA between 59 and a 70% after incubation at 37 °C for 60 min in a buffer solution of pH 5. Part of the OTA adsorption abilities of the yeasts rely on the structure of their walls. The cell wall is a bi-layered structure, mainly made up of two glucans, named β-(1,3)-D-glucan and β-(1,6)-D-glucan. The distribution between these two glucans plays an important role in the efficacy of the binding capacity [75]. Linked to these glucans are the cell wall proteins, called mannoproteins. Mannoproteins are composed mainly of carbohydrates, from which mannan oligosaccharides play an important role in OTA adsorption to the cell surface [19]. Both β-glucans and mannan oligosaccharides provide the cell wall with weak hydrogen and van der Waals non-covalent bonds to OTA. Due to the nature of these bonds, OTA adsorption is a fast and reversible mechanism [81,82].

#### 3.3.3. Biodegradation of Ochratoxin A by Lactic Acid Bacteria

**Figure 2:**
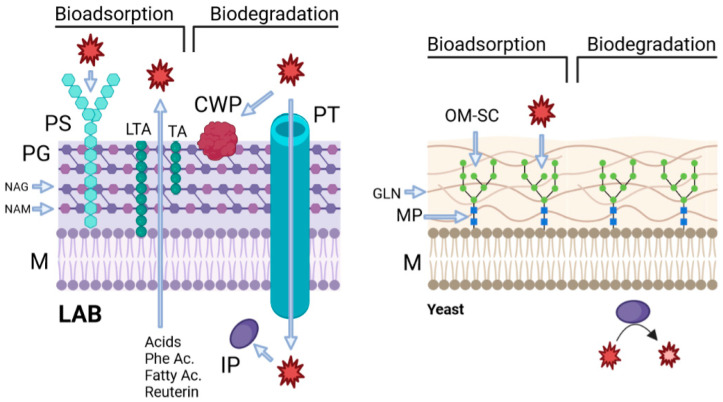
OTA adsorption and degradation mechanisms of LAB and yeasts found in coffee. Created in Biorender.com. M = membrane; PG = peptidoglycan; PS = polysaccharides; NAG = N-acetyl glucosamine; NAM = N-acetyl muramic acid; LTA = lipoteichoic acids; TA = teichoic acids; CWP = cell wall protein; PT = peptide transporter; IP = intracellular peptidase; MP = mannoproteins; GLN = glucan; OM-SC = mannan oligosaccharides.

Biodegradation is the second most widely known process for reduction in OTA by LAB after adsorption. The mechanism usually involves enzymatic reactions that will lead to the breakdown of OTA into other potential compounds. Biodegradation poses two disadvantages compared to adsorption: it is more time-consuming and might produce harmful metabolites [35]. Also, although rarely, some studies describe bacteria reducing OTA via both degradation and adsorption at the same time [83,84]. LAB produce several enzymes that can degrade mycotoxins to other compounds. These enzymes can be proteolytic enzymes, such as cell wall-bound proteinase, that hydrolyse proteins into polypeptides and peptide transporters, which transfer the peptides into the cells, and intracellular peptidases degrade the transferred peptides into amino acids, as represented in Figure 2 [76].

Du et al. (2021) [85] studied the potential of Tibetan Kefir Grains, composed mainly of *Lactiplantibacillus kefiranofaciens* and *Kazachstania unisporus* AC-2, to detoxify dairy products. In their experiments on liquid media, they discovered that 75% of the OTA was bound to the cell wall and only 25% of the OTA was degraded, giving ochratoxin alpha (Otα) and phenylalanine (Phe) as the main degradation products. Similarly, Zhang et al. (2022) analyzed the ability to remove OTA of *Bacillus velezensis* E2 on *A. westerdijkiae* fc-1 on a liquid medium. Their results suggested that the mechanisms responsible for 98.4% of the OTA by the cell-free supernatant might be of enzymatic origin. The second reduction mechanism analyzed in this study was alkaline hydrolysis, which consists of the opening of the lactone ring of OTA under alkaline conditions (pH 8–12) and converting the OTA into open-ring OTA (OP-OTA). Both the enzymatic and the alkaline hydrolysis theories were supported by the identification of degradation products Otα and OP-Otα [77].

Einloft et al. (2017) isolated two strains of *Bacillus* genus, *B. amyloliquefaciens* RP103 and *B. subtilis* RP242. These strains showed high OTA inhibition when confronted to *A. westerdijkiae* in a coffee-based medium. Specifically, *B. amylolicheniformis* showed an OTA concentration reduction by 92%, which might be linked to the degradation by cells and/or cell-free metabolites [64]. Additionally, Shi et al. (2014) isolated, from elk droppings, a strain of *B. subtilis*, whose cell-free supernatant degraded 97.6% of OTA during incubation with *A. ochraceus* [83]. As described in Table 5, certain food commodities affected by the same ochratoxigenic fungi as coffee have shown the successful isolation of bacteria with the ability to degrade OTA. For example, *Pediococcus parvulus*, isolated from Douro wines, degraded 90% of OTA into Otα [24]. Also, two bacteria isolated from vineyard soil of the genus *Acinetobacter* sp. reduced OTA concentration by 80% [26].

#### 3.3.4. Biodegradation of Ochratoxin A by Yeasts

To the best of our knowledge, there are no publications of yeasts, isolated from coffee or used in coffee media or coffee beans, with OTA degradation ability. There is very little information about the purified and characterized enzymes involved in OTA degradation. Most of the purified enzymes capable of degrading OTA are produced by yeast strains isolated from soil samples [86]. For example, the enzyme carboxypeptidase Y (CPY) purified from *S. cerevisiae* has been notified to covert 52% of OTA present in the assay into OTα [59]. However, there are some successful yeasts isolated from grapes presented in Table 5, such as *Yarrowia lipolytica* Y-2, with the ability to degrade 97.5% of OTA into OTα through intracellular enzymes [87].

**Table 5:** Culture or enzymes of bacteria or yeasts with the ability of OTA degradation on food commodities similar to coffee.

Micro-Organism or Enzymes	OTA Concentration	Removal (%)	Incubation Time/Condition	Degradation Products	Reference
*Acinetobacter calcoaceticus* 396.1	1 µg/mL	82	6 d in liquid medium	OTα	[26]
*Acinetobacter* sp.	1 µg/mL	91	6 d in liquid medium	OTα	[26]
*Pediococcus parvulus*	1 µg/mL	90	25 h in liquid medium	OTα	[24]
*Yarrowia lipolytica* intracellular enzymes	1 µg/mL	97.5	1 d in liquid medium	OTα	[87]

#### 3.3.5. Biodegradation of Ochratoxin A by Non-Mycotoxigenic Fungi

There is very little information about the purified and characterized enzymes from fungi involved in OTA degradation. *A. niger* has been a source of many enzymes such as lipase, protease A, prolyve PAC, and a cell-free crude enzyme preparation. Generally, there are two types of reactions of OTA with these enzymes: hydrolysis of the amide bond, or hydrolysis of the lactone ring [88]. An example of the OTA-degrading properties of a UV-mutated strain of *A. niger* is analyzed in the work of Zou et al. (2022) [89]. In vitro, at a pH 8 and 30 °C, this strain can degrade 89.4% of OTA to non-toxic derivates such as Otα and a new metabolite of OTA degradation identified as P1. It has also been tested on contaminated wheat bran, where it degraded 59.8% of OTA.

### 3.4. Physical Strategies for Reduction in Ochratoxin Reduction in A in Coffee

There are various physical strategies such as extrusion, ultraviolet (UV) and gamma radiation, high-pressure, ultrasound, and cold plasma to detoxify mycotoxins in coffee [88]. These strategies can be applied at different stages of the coffee chain, after harvest, before fermentation, or after roasting. Until now, cold plasma has been used on roasted coffee, inhibiting the growth of OTA-producing fungi and reducing 50% of the OTA content. This technique does not affect the nutritional and sensory properties of coffee, but it can generate reactive species such as O_2_, O_3_, OH, NO, and NO_2_ [90].

Torrefaction can reduce OTA in coffee, although the reduction is normally heterogeneous, from 12 to 96% [33]. Optimal conditions such as lower initial level of OTA, longer roasting time, and roasting method (for example, a rotating cylinder presents better reduction in OTA levels than a fluidized bed) could improve the efficiency of the roasting process, reaching OTA levels below the threshold set by the EC [91]. Torrefaction process has been reported to degrade OTA into less cytotoxic products, 14-decarboxy-OTA and 14-(R)-OTA, through decarboxylation and isomerization reactions, respectively [88].

Ultrasounds reduce the OTA in coffee through inactivation of filamentous fungi and through a direct degradation of the OTA. This method has been used before fermentation without affecting the quality of coffee. Gamma irradiation and high-pressure processing (HPP) are proposed as well because they would not have any change on the organoleptic properties of coffee. The efficacy of gamma irradiation in reducing the OTA concentration is directly correlated to a_w_. A a_w_ of 0.82, 0.65, and 0.59 reduce 90%, 9%, and 5% of the OTA concentration, respectively. HPP at 400 MPa or higher inhibits fungal growth but does not reduce the OTA concentration. Additionally, HPP increases the content of beta-aminobutyric acid after 50 days of storage, which improves the nutritional value of coffee [3].

Byun et al. (2020) analyzed the effect of UV-C irradiation on *A. flavus* and *A. parasiticus* spores’ survival on roasted coffee beans. The results confirmed that UV-C was effective in preventing the contamination of roasted coffee beans by *A. flavus*, but not sufficiently effective with *A. parasiticus*. Regarding the effect on roasted coffee beans quality, UV-C irradiation modified the Hunter color, lowered the pH, and decreased the hedonic points by enhancing sourness [92]. Although the treatment of 3 h with UV-C has been shown to reduce OTA contamination by more than 80% in rice [93], there are no published studies investigating the effects of UV-C on degrading OTA in coffee.

### 3.5. Chemical Strategies for Reduction in Ochratoxin A in Coffee

Chemical methods for OTA detoxification include bases, oxidizing agents, organic acids, and other agents. Some studies on cocoa shells or grapes use potassium carbonate, reaching a reduction in OTA by 95 and 50%, respectively [94]. Also, sodium hydrosulfite applied to black pepper has reduced OTA by 96% [95]. These two bases, together with acids such as lactic acid, citric acid, and acetic acid, were effective in reducing OTA in food [96]. However, the use of these chemicals in food is regulated due to safety and nutritional concerns, and the number of studies on OTA detoxification through chemical methods in coffee is very scarce [88]. The treatment with gaseous ozone in coffee was investigated for its efficacy to reduce ochratoxigenic fungi and OTA presence in Arabica green coffee beans contaminated with *A. carbonarius*, *A. westerdijkiae*, and *A. ochraceus* under experimental conditions mimicking storage environments. A maximum reduction by 26% of OTA was observed after storage for 12 days at 0.95 a_w_ and 30 °C [12]. However, its potential application is only relevant for stored coffee after fermentation and during medium-term storage and transport.

## 4. Conclusions

There are many complementary approaches to reduce OTA occurrence in coffee. Prevention and decontamination may be promoted by the use of BCAs. This solution emerges as the most promising approach, effectively reducing coffee losses by proactively preventing OTA accumulation. This review has highlighted the diversity of the micro-organisms tested. Some were tested as whole cultures, while others were tested as supernatants. Those promising results were most often limited to in vitro conditions.

The results summarized in this review highlight the link between the biocontrol of toxigenic fungi and adsorption/degradation of mycotoxin strategies, exposing the need for further tests to recognize the mechanisms of OTA reduction. This review of biocontrol strategies reveals a lack of in vivo tests and proposes the test performed by De Melo Pereira et al. (2016) [65], using *P. fermentans* LPBYB13, as the most advanced study towards the potential application of BCAs during coffee fermentation.

## 5. Future Research Needs

BCAs in coffee have great potential to prevent OTA occurrence. However, the heterogeneity of the tested conditions and the micro-organisms studied has led to difficulties in comparing results. Consequently, we suggest insights for future studies in coffee biocontrol.

Improved data input in future articles is required, particularly for the coffee species (Robusta or Arabica), process (wet or dry), fungi related to this contamination or harvesting year, inoculum concentration (in CFU/mL), and analysis of OTA degradation by-products. Also, there is a very low number of studies on soluble coffee, which is alarming since the EC’s maximum limit for OTA is lower for soluble coffee than for roasted coffee.

The candidates, the BCAs with OTA presentation potential, should be tested in vivo in coffee beans. The protocol established by De Melo Pereira et al. (2016), which evaluates the biocontrol efficacy of diverse micro-organisms in coffee beans, serves as a valuable benchmark. A marginal enhancement could involve the incorporation of multiple ochratoxigenic species to ascertain the biocontrol agent’s potential in diminishing the OTA concentration [65].

To date, there is no published data about the combination of various biocontrol agents isolated from coffee. As De Melo Pereira et al. (2020) pointed out in their review, the combined use of LAB and yeasts in the coffee chain holds a promising research line, since both micro-organisms are part of the innate coffee microbiota, and their role in coffee fermentation is known to improve organoleptic quality and control mycotoxigenic fungi [18].

By applying these recommendations, we could have comparable data between the different BCAs or adsorption/degradation agents. The future of those methods is the development and formulation of inoculums of OTA BCAs or adsorption/degradation agents to optimize efficacy and provide solutions to the coffee chain. The demand for this kind of biological agents will further increase in the future. Additionally, there is a research gap in the application of chemical agents to reduce OTA in coffee other than ozone.

## Figures and Tables

**Table 1:** Optimal conditions of growth, sporulation, and Ochratoxin A (OTA) production for the most ochratoxigenic fungi in coffee.

Species	Optimal Growth		Optimal Spore Germination		Optimal OTA Production		References
	T (°C)	a_w_	T (°C)	a_w_	T (°C)	a_w_	
*A. ochraceus*	24–31	0.95–0.99	20–30	0.95–0.99	25–30	0.98	[10,11]
*A. westerdijkiae*	20–30	0.95–0.99	20–30	0.95–0.99	28	0.99	[12]
*A. steynii*	28–32	0.95–0.99	20–30	0.95–0.99	28	0.99	[13]
*A. carbonarius*	32–37	0.95–0.99	32–35	0.95	15–25	0.96–0.98	[14,15]

## Data Availability

No new data were created or analyzed in this study. Data sharing is not applicable to this article.
